# Mitochondria-dependent phase separation of disease-relevant proteins drives pathological features of age-related macular degeneration

**DOI:** 10.1172/jci.insight.142254

**Published:** 2021-05-10

**Authors:** Nilsa La Cunza, Li Xuan Tan, Thushara Thamban, Colin J. Germer, Gurugirijha Rathnasamy, Kimberly A. Toops, Aparna Lakkaraju

**Affiliations:** 1 Department of Ophthalmology, School of Medicine, and; 2 Pharmaceutical Sciences and Pharmacogenomics Graduate Program, Graduate Division, University of California, San Francisco, California, USA.; 3 Department of Ophthalmology and Visual Sciences, University of Wisconsin-Madison, Madison, Wisconsin, USA.; 4 Department of Anatomy, School of Medicine, University of California, San Francisco, California, USA.

**Keywords:** Ophthalmology, Cholesterol, Complement, Retinopathy

## Abstract

Age-related macular degeneration (AMD) damages the retinal pigment epithelium (RPE), the tissue that safeguards photoreceptor health, leading to irreversible vision loss. Polymorphisms in cholesterol and complement genes are implicated in AMD, yet mechanisms linking risk variants to RPE injury remain unclear. We sought to determine how allelic variants in the apolipoprotein E cholesterol transporter modulate RPE homeostasis and function. Using live-cell imaging, we show that inefficient cholesterol transport by the AMD risk-associated ApoE2 increases RPE ceramide, leading to autophagic defects and complement-mediated mitochondrial damage. Mitochondrial injury drives redox state–sensitive cysteine-mediated phase separation of ApoE2, forming biomolecular condensates that could nucleate drusen. The protective ApoE4 isoform lacks these cysteines and is resistant to phase separation and condensate formation. In *Abca^–/–^* Stargardt macular degeneration mice, mitochondrial dysfunction induces liquid-liquid phase separation of p62/SQSTM1, a multifunctional protein that regulates autophagy. Drugs that decrease RPE cholesterol or ceramide prevent mitochondrial injury and phase separation in vitro and in vivo. In AMD donor RPE, mitochondrial fragmentation correlates with ApoE and p62 condensates. Our studies demonstrate that major AMD genetic and biological risk pathways converge upon RPE mitochondria, and identify mitochondrial stress-mediated protein phase separation as an important pathogenic mechanism and promising therapeutic target in AMD.

## Graphical abstract

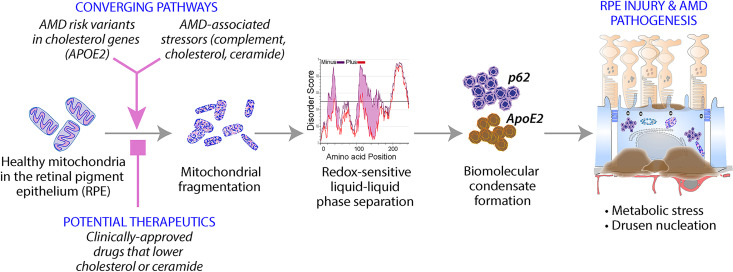

## Introduction

Age-related macular degeneration (AMD), a complex neurodegenerative disease that destroys central high-resolution vision, is the most common cause of permanent blindness in older adults. Global AMD prevalence is projected to exceed 200 million by the year 2040 largely due to lack of disease-modifying therapies for non-neovascular or “dry” AMD, which affects the majority of patients (1, 2). The primary site of injury in AMD is the retinal pigment epithelium (RPE), a monolayer of postmitotic polarized cells that performs numerous functions essential for photoreceptor health and for vision (3, 4). In early AMD, RPE dysfunction is accompanied by the deposition of sub-RPE and subretinal lipid-protein aggregates called drusen (5–7). Clinically, RPE abnormalities and drusen location, size, and morphology are strong predictors of AMD progression (1). Polymorphisms in over 50 independent genes involved in regulating complement activation, cholesterol homeostasis, oxidative stress, and extracellular matrix remodeling are associated with AMD. Yet, we have limited insight into the mechanisms by which AMD-associated variants promote RPE injury and drusen formation (7–9).

Of the cholesterol regulatory genes implicated in AMD, the cholesterol transporter *APOE* is especially enigmatic because humans are the only species that express *APOE* allelic variants, which show reversed risk associations between AMD and Alzheimer’s disease. *APOE4* significantly reduces the risk of developing AMD and *APOE2* is associated with increased susceptibility (8, 10–12), whereas the opposite is true for Alzheimer’s disease (13, 14). At least 3 lines of evidence show that ApoE and cholesterol are major players in AMD pathogenesis: the RPE is the primary biosynthetic source of ApoE in the retina; ApoE secretion by polarized RPE monolayers is increased upon complement exposure (6, 15, 16); and drusen from AMD eyes are rich in cholesterol and ApoE (5). ApoE transports cholesterol in association with ABCA1, another protein implicated in AMD, and RPE-specific deletion of *ABCA1* in mice causes RPE cholesterol storage and progressive RPE atrophy, photoreceptor loss, and retinal degeneration (17). These studies suggest that isoform-specific functions of ApoE could have a major impact on the RPE homeostasis. However, precisely how *APOE* variants influence RPE health and drusen formation and how this impacts disease pathogenesis are critical, unresolved questions in AMD biology.

Work from our group has identified complex multilayered interactions between the cholesterol and complement pathways in derailing RPE homeostasis in models of macular degeneration. We have shown that excess RPE cholesterol in the *Abca4^–/–^* mouse model of Stargardt macular degeneration leads to a secondary accumulation of ceramide, which in turn compromises mechanisms that protect the RPE from complement attack. Sustained complement activation eventually causes mitochondrial injury and metabolic deficits in the RPE (18, 19). Of note, although mitochondrial abnormalities have been documented in the RPE of patients with AMD (20–22), mechanisms connecting loss of mitochondrial integrity with specific features of AMD pathology have yet to be identified.

Here, we sought to investigate how ApoE isoforms regulate cholesterol transport and mitochondrial integrity and determine how this could contribute to drusen biogenesis. Using high-speed, live-cell imaging of polarized adult primary RPE monolayers, *Abca4^–/–^* mice, and human AMD donor tissues, we show that efficient cholesterol transport in RPE expressing the nonrisk ApoE3 or the protective ApoE4 isoforms preserves critical homeostatic functions such as autophagy despite cellular stress. In contrast, ApoE2-expressing RPE accumulate cholesterol, making the RPE susceptible to complement-mediated mitochondrial fragmentation. Our data identify an exciting mechanism of redox-mediated liquid-liquid phase separation in the RPE. We show that fragmented mitochondria promote ApoE2 phase separation as a result of thiol oxidation of cysteines at 112 and 158, resulting in the formation of biomolecular condensates as potential drusen precursors. The AMD protective ApoE4, with arginines at both positions, is resistant to phase separation, providing a biophysical basis for ApoE risk associations in AMD. RPE mitochondrial injury drives redox state–dependent phase separation of other proteins with reactive cysteines, notably the multifunctional protein p62/SQSTM1. As p62 regulates autophagy, the proteasome, and the antioxidant stress response, this would have far-reaching consequences for RPE health. Drugs that decrease RPE cholesterol or ceramide prevent mitochondrial injury and limit the formation of biomolecular condensates in *Abca4^–/–^* mice. Using human tissue as an ex vivo model, we observed significantly more mitochondrial fragmentation and larger ApoE and p62 condensates in AMD donor RPE.

This study integrates genetic and biological risk pathways with poorly understood features of AMD: cholesterol and complement dysregulation, mitochondrial injury, and drusen biogenesis. Our data provide nuanced insight into how a key genetic risk variant mediates crosstalk between lipid and complement pathways to disrupt critical metabolic functions and identify mitochondria as a mediator of liquid-liquid phase separation of proteins implicated in AMD. Our studies also suggest that targeting upstream mechanisms that preserve redox homeostasis could be a promising therapeutic approach to limit phase separation and safeguard RPE health.

## Results

### Isoform-specific dynamic ApoE trafficking alleviates pathological cholesterol accumulation in the RPE.

In the human *APOE* gene, 2 polymorphisms (rs7412 and rs429358) result in cysteine/arginine interchanges at positions 112 and 158 in the ApoE protein: ApoE2 has 2 cysteines, ApoE3 has a cysteine and an arginine, and ApoE4 has 2 arginines (Supplemental Figure 1A; supplemental material available online with this article; https://doi.org/10.1172/jci.insight.142254DS1). The single amino acid changes between ApoE2, ApoE3, and ApoE4 substantially alter protein structure and function, including oligomer formation, and binding to lipids and receptors (23–27). *APOE3* is the most prevalent allele and *APOE2* the least (~79% global allele frequency for *APOE3* compared with approximately 7% for *APOE2* and 14% for *APOE4*) (28).

**Figure 1:**
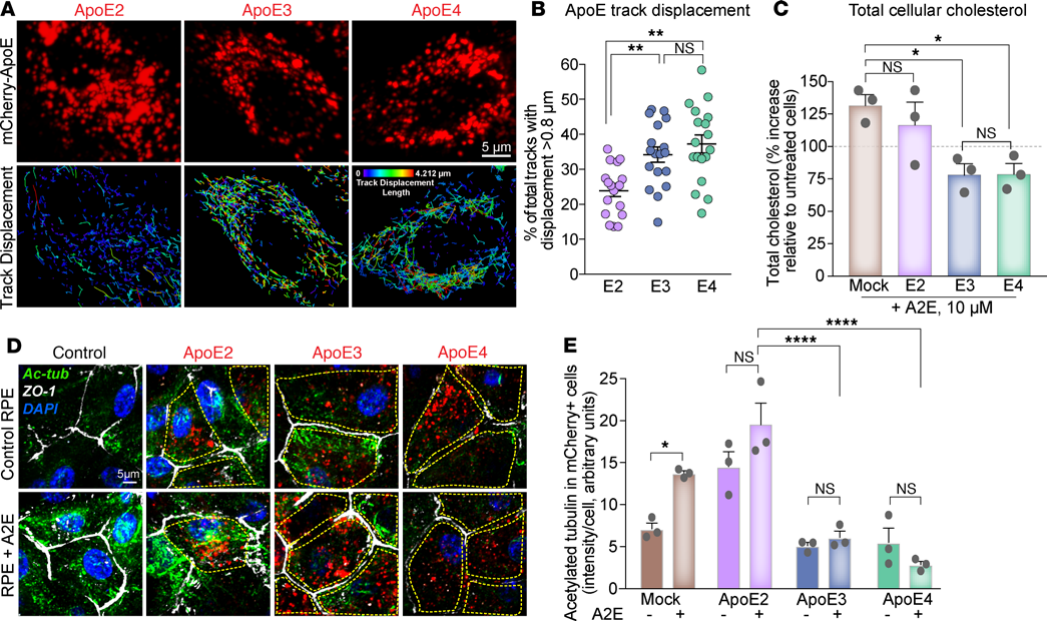
ApoE isoform-specific differences in regulating RPE cholesterol transport and microtubule dynamics. (**A**) Stills from live imaging of mCherry-tagged human ApoE2, ApoE3, or ApoE4 (top panel, red) in primary RPE cultures. Tracks of individual ApoE vesicles (lower panel). Color bar shows displacement of individual tracks from short (cooler colors) to long (warmer colors); range, 0.0 μm to 4.212 μm. (**B**) Percentage of total ApoE tracks with displacement > 0.8 μm in RPE expressing ApoE2, ApoE3, or ApoE4. Mean ± SEM, *n* >18 cells per condition from 3 independent experiments. **P < 0.005. (**C**) Endogenous cholesterol content in mock-transfected or ApoE2-, ApoE3-, or ApoE4-expressing RPE treated with A2E. Mean ± SEM from 3 independent experiments, 3 replicates/experiment. (**D**) Representative images and quantitation of acetylated tubulin (green) immunostaining in mock-transfected or ApoE2-, ApoE3-, or ApoE4-expressing RPE treated with or without A2E. Cell boundaries are demarcated by zonula occludens 1 (ZO-1) (white) and nuclei are labeled with DAPI (blue). Cells expressing mCherry-ApoE (red) are outlined in yellow. (**E**) Quantification of acetylated tubulin intensity in mCherry-expressing cells. Mean ± SEM, *n* = 10 cells/condition. (**B**, **C**, and **E**) One-way ANOVA with Bonferroni’s posttest. *P < 0.01, **P < 0.001, ****P < 0.0001. See also Supplemental Figure 1.

To determine how ApoE isoforms modulate steady-state cholesterol levels in healthy and stressed RPE, we first performed high-speed, live-cell imaging of primary RPE expressing mCherry-tagged ApoE2, ApoE3, or ApoE4 (Supplemental Figure 1A). We observed comparable transfection efficiencies and expression of human ApoE fusion proteins measured by immunoblotting and single-cell mCherry fluorescence (Supplemental Figure 1, B and C). Four-dimensional analysis of ApoE vesicle trajectories showed that ApoE3 and ApoE4 vesicles exhibited long-range, directed movements (large displacements) compared with ApoE2 (Figure 1, A and B), likely due to structural and conformational differences between the isoforms. Intermolecular disulfide bonds between cysteines at positions 112 and 158 in ApoE2 (Supplemental Figure 1A) could induce the formation of multimers that are retained within the cell, as reported for macrophages and adipocytes (29, 30). Additionally, ApoE4 has a closed conformation due to domain interactions via salt bridges between the N-terminal receptor binding domain and the C-terminal lipid-binding domain, whereas ApoE2 adopts the most open conformation and ApoE3 an intermediate conformation (31). Thus, the more compact, dynamic structures of ApoE3 and ApoE4 relative to ApoE2 would enable efficient intracellular trafficking.

Pathological accumulation of lipofuscin bisretinoids, vitamin A metabolites of the visual cycle, is associated with inherited and age-related macular degeneration (32). Because bisretinoids such as A2E cause a secondary accumulation of cholesterol in the RPE (18, 19, 33, 34), we asked how ApoE isoforms would impact the ability of the RPE to deal with excess cholesterol. Biochemical measurements showed that the expression of ApoE3 or ApoE4, but not ApoE2, prevented A2E-induced increase in total cell cholesterol (Figure 1C).

Bidirectional lipoprotein secretion by polarized RPE shuttles free and esterified cholesterol into and out of the retina (35). We used filipin (33) and BODIPY 493/503 (36), respectively, to measure free cholesterol and neutral lipids in RPE expressing ApoE2, ApoE3, or ApoE4. Compared with ApoE3-expressing RPE (ApoE3-RPE) and ApoE4-RPE, ApoE2-RPE had significantly more lipid droplets (Supplemental Figure 1, D and E) and free cholesterol (Supplemental Figure 1F), reflecting their differential trafficking dynamics.

Cell cholesterol modulates organelle biogenesis and trafficking by regulating membrane dynamics and association with microtubules (37). We have shown that lipofuscin-mediated cholesterol storage in the RPE leads to aberrant activation of acid sphingomyelinase (ASMase), the enzyme that hydrolyzes sphingomyelin to ceramide. Increased ceramide results in the accumulation of stable acetylated microtubules, which interfere with autophagosome trafficking, endosome biogenesis, and lysosome localization (18, 19, 33). Immunostaining of RPE expressing ApoE2, ApoE3, and ApoE4 for acetylated tubulin showed that the expression of either ApoE3 or ApoE4 rescues the A2E-mediated increase in tubulin acetylation in the RPE, whereas ApoE2 did not (Figure 1, D and E).

Taken together, these studies indicate that ApoE2 exhibits significant defects in intracellular trafficking and cholesterol transport, likely due to cysteines at 112 and 158, which promote intracellular retention (29–31, 38). Further, dynamic long-range trafficking of ApoE3 and ApoE4 is essential for maintaining cholesterol homeostasis and microtubule-based trafficking in the RPE.

### ApoE2 aggravates autophagic defects induced by lipofuscin bisretinoids.

To understand how ApoE isoform-specific cholesterol transport impacts critical RPE functions, we investigated autophagy, an evolutionarily conserved mechanism responsible for degrading and recycling cellular debris. Lipofuscin-induced cholesterol accumulation and disruption of microtubule-based transport interfere with autophagy in *Abca4^–/–^* mice and in primary RPE cultures with A2E (33). These autophagic defects manifest as decreased autophagosome biogenesis (decreased LC3B lipidation) and trafficking and impaired autophagic flux.

**Figure 2:**
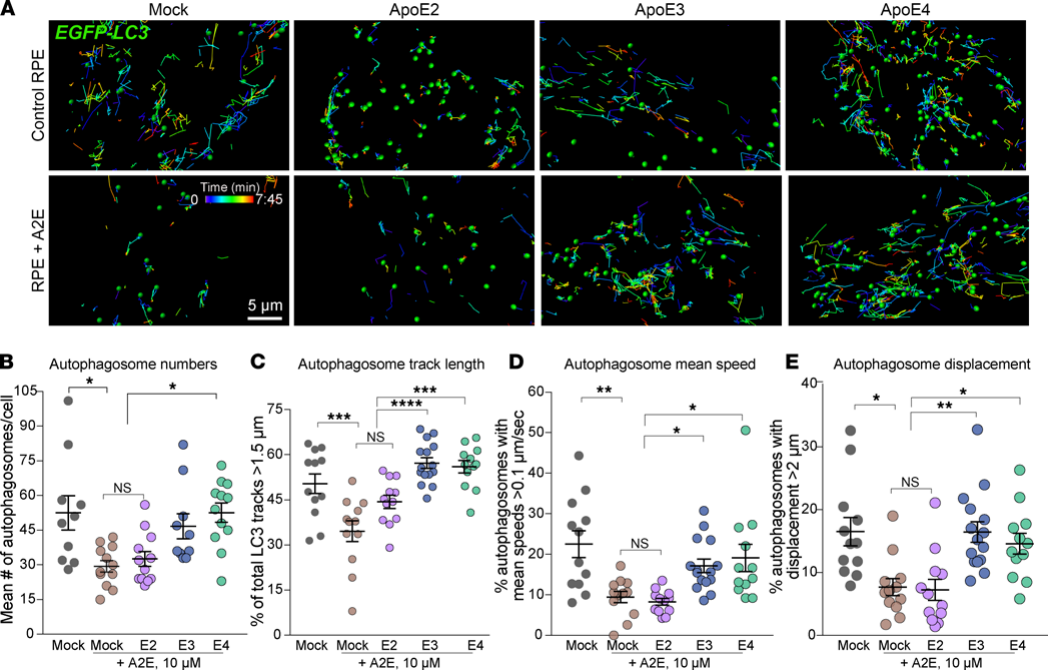
ApoE2 aggravates autophagic defects induced by lipofuscin bisretinoids. (**A**) Stills from spots and tracks analyses of live imaging of EGFP-LC3 autophagosome trafficking in mock-transfected RPE or RPE expressing mCherry-tagged ApoE2, ApoE3, or ApoE4 treated with or without A2E. (**B**) Average number of EGFP-LC3 autophagosomes per cell. (**C**) Percentage of total EGFP-LC3 tracks longer than 1.5 μm. (**D**) Percentage of autophagosomes with mean speeds > 0.1 μm/s. (**E**) Percentage of autophagosomes with displacement > 2 μm in mock-transfected or ApoE2-, ApoE3-, or ApoE4-expressing RPE treated with or without A2E. Mean ± SEM, *n* > 13 cells per condition. *P < 0.05, **P < 0.005, ***P < 0.001, ****P < 0.0001. (**B**–**E**) One-way ANOVA with Bonferroni’s posttest. See also Supplemental Figure 2 and Supplemental Videos 1–8.

We performed high-speed live-cell imaging of EGFP-LC3–labeled autophagosomes in primary RPE expressing ApoE2, ApoE3, or ApoE4 (Figure 2A, Supplemental Figure 2A, and Supplemental Videos 1–8). As expected, A2E decreased autophagosome biogenesis in mock-transfected RPE (33). The expression of ApoE3 or ApoE4, but not ApoE2, increased autophagosome numbers in RPE with A2E, comparable to control levels (Figure 2B). Analysis of LC3 trafficking showed that autophagosomes in RPE with A2E had predominantly shorter tracks, with significantly lower mean speeds and displacements (mock-transfected control vs. mock-A2E; Figure 2, C–E; and Supplemental Videos 1 and 2). RPE expressing ApoE3 or ApoE4 were resistant to A2E-induced disruption of autophagosome trafficking, whereas ApoE2 expression was ineffective (Figure 2, C–E; Supplemental Figure 2, B–G; and Supplemental Videos 3–8). These data suggest that improved microtubule dynamics in ApoE3-RPE and ApoE4-RPE are essential for stressed RPE to maintain efficient autophagy.

Decreased autophagosome biogenesis and autophagic flux have been reported in AMD donor RPE (20, 39). Our data identify RPE cholesterol as an important determinant of autophagy and suggest that protective variants of AMD-associated cholesterol pathway genes could modulate RPE health by preventing autophagic deficits.

### ApoE2 increases RPE susceptibility to complement-mediated mitochondrial injury.

**Figure 3:**
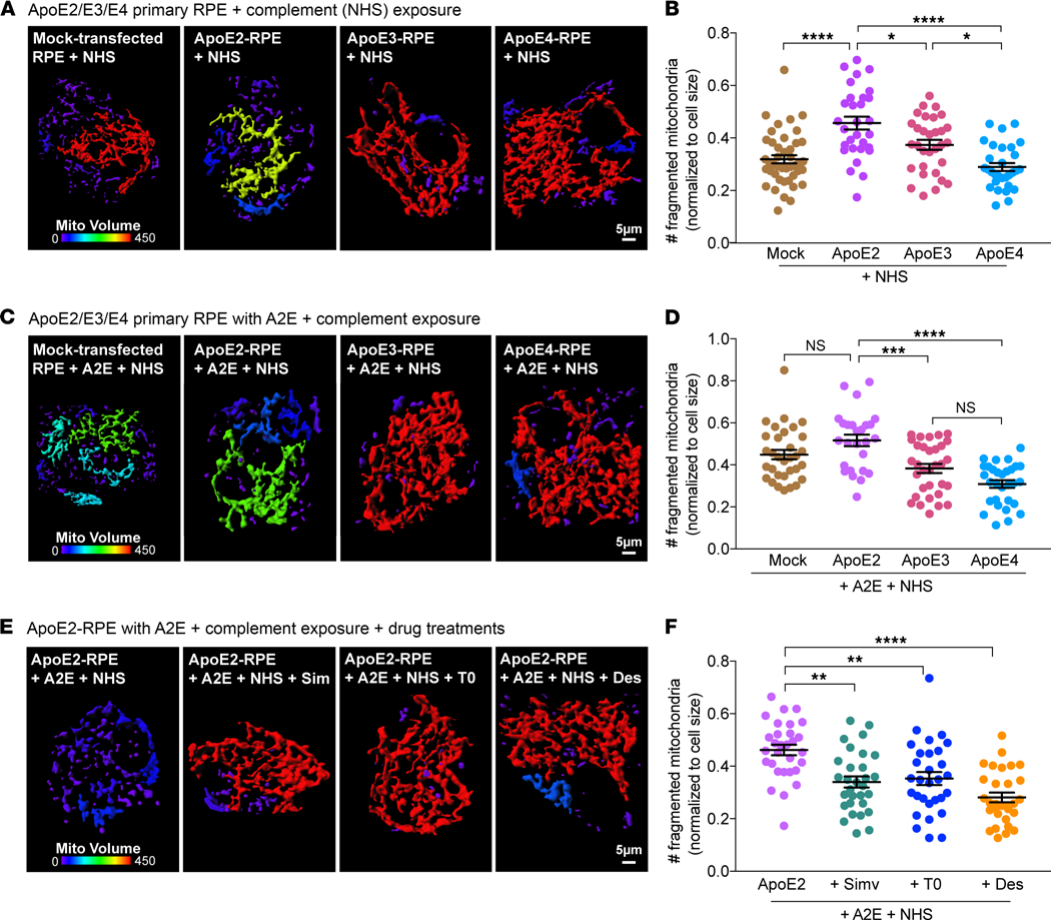
ApoE2 exacerbates complement-induced mitochondrial fragmentation in the RPE. (**A**) 3D reconstruction of mitochondrial volumes from live imaging of MitoTracker in mock-transfected RPE or RPE expressing mCherry-tagged ApoE2, ApoE3, or ApoE4 exposed to 10% NHS to induce complement attack. Cooler colors in the color bar indicate increasing mitochondrial fragmentation. (**B**) Number of fragmented mitochondria after NHS exposure. (**C**) Mitochondrial volumes as in **A** in RPE treated with A2E prior to NHS exposure. (**D**) Quantification of mitochondrial fragments as in **B**. (**E**) Mitochondrial volumes as in **A** in ApoE2-expressing RPE with A2E exposed to NHS and treated with simvastatin (5 μM, 16 h), T0901317 (1 μM, 16 h), or desipramine (10 μM, 3 h) prior to imaging. (**F**) Quantification of mitochondrial fragments as in **B**. Mean ± SEM, *P < 0.05, **P < 0.005, ***P < 0.0005, ****P < 0.0001. *n* = 30 cells (**A**), 26 cells (**B**), and 30 cells (**C**) per condition; 1-way ANOVA with Bonferroni’s posttest. See also Supplemental Figure 3.

Abnormal activation of the alternative complement pathway is strongly associated with AMD. The RPE, which forms the outer blood-retinal barrier, is the first line of defense against uncontrolled complement activation. We have reported that in models of macular degeneration, acetylated microtubules prevent recycling of the complement-regulatory protein CD59 to the plasma membrane and inhibit membrane repair by lysosome exocytosis. This enables uncontrolled assembly of the C5b-9 terminal membrane attack complex pore on the cell membrane, and the resulting increase in intracellular calcium causes mitochondrial fragmentation and oxidative stress in the RPE (19). To understand how AMD-associated genes (e.g., ApoE) interact with biological risks (e.g., lipofuscin, cholesterol, complement) to promote mitochondrial injury, we performed live-cell imaging of RPE mitochondrial networks before and after exposure to normal human serum (NHS) as a source of complement (19). Reconstruction of mitochondrial volumes showed highly integrated mitochondria in mock-transfected and ApoE3- or ApoE4-expressing RPE, in agreement with our previous finding (19) that healthy RPE monolayers are resistant to complement attack. In contrast, mitochondria in RPE expressing ApoE2 were fragmented in response to NHS (Figure 3, A and B), indicating that complement-regulatory mechanisms were impaired in ApoE2-RPE, likely due to increased tubulin acetylation. As expected, A2E rendered mock-transfected RPE vulnerable to NHS. In contrast to ApoE2-RPE, the expression of ApoE3 or ApoE4 suppressed mitochondrial fragmentation in RPE with A2E after NHS exposure (Figure 3, B and C). Thus, better cholesterol homeostasis and decreased ceramide in the RPE help maintain dynamic microtubule trafficking required to combat complement activation.

If complement-mediated mitochondrial fragmentation is a result of excess cholesterol activating ASMase, which in turn increases ceramide to induce tubulin acetylation, then drugs that decrease cholesterol or inhibit ASMase (19, 33) should maintain RPE mitochondrial integrity after complement attack. In support of this hypothesis, inhibiting cholesterol biosynthesis with the lipophilic statin simvastatin, increasing cholesterol efflux with the liver X receptor (LXR) agonist T0901317, or inhibiting ASMase with desipramine all decreased complement-induced mitochondrial damage in both mock-transfected (Supplemental Figure 3, A and B) and ApoE2-expressing RPE with A2E after exposure to NHS (Figure 3, E and F). Taken together, these data demonstrate that genetic variants or pharmacological approaches that limit excess cholesterol safeguard the RPE from complement attack and protect mitochondrial integrity.

### ApoE exhibits liquid-like behavior and undergoes phase separation in the RPE.

The accumulation of ApoE- and cholesterol-rich aggregates called drusen in the subretinal and sub-RPE space is a hallmark of AMD. Clinically, drusen size, shape, and abundance correlate with AMD progression. Although studies suggest that the RPE is the minimal requirement for drusen formation and complement exposure increases ApoE secretion by RPE cells in culture (16, 40, 41), molecular mechanisms responsible for drusen biogenesis are unknown. To determine how AMD-associated stressors can nucleate drusen within the RPE, we hypothesized that mitochondrial dysfunction in the RPE could induce ApoE to undergo liquid-liquid phase separation to form biomolecular condensates (42–45). Proteins that undergo phase separation are characterized by intrinsically disordered regions (IDRs) that enable them to engage in low-affinity interactions with one another (46). Because ApoE isoforms had IDRs (Supplemental Figure 4, A–C) (47), it is plausible that aberrant phase transitions could drive ApoE condensate formation in the RPE as precursors to drusen.

**Figure 4:**
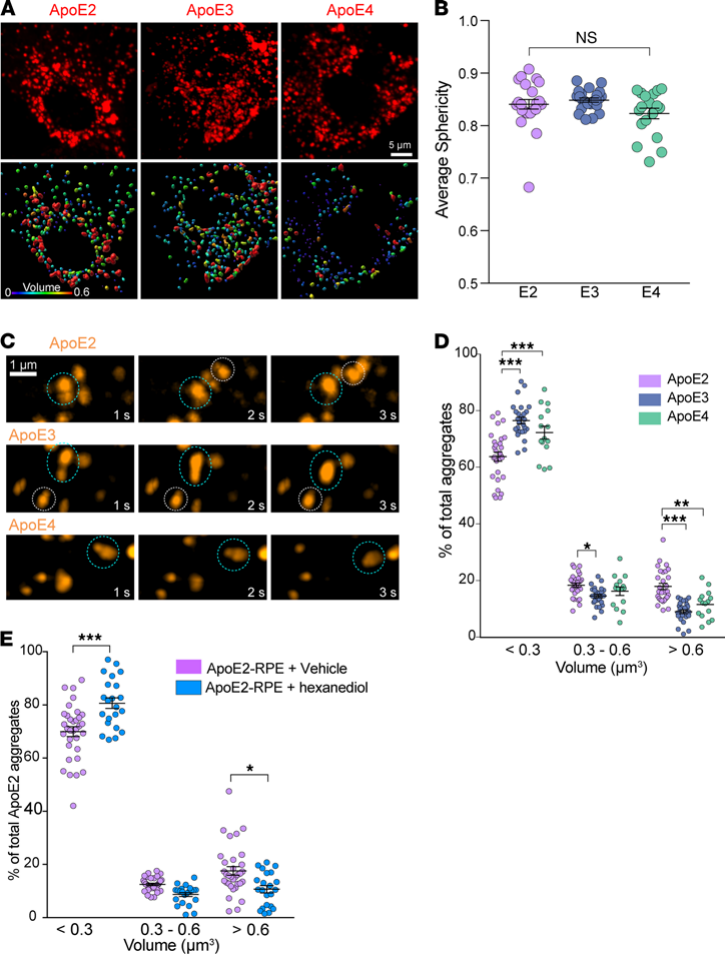
ApoE exhibits liquid-like behavior in the RPE. (**A**) Representative stills (top panel) and 3D volume reconstructions (lower panel) from live imaging of intracellular mCherry-tagged ApoE2, ApoE3, or APoE4 in the RPE. Warmer colors indicate larger volumes. (**B**) Average sphericity of ApoE condensates in RPE expressing ApoE2, ApoE3, or ApoE4. Mean ± SEM, *n* = 17–23 cells per condition, n.s. (**C**) Time-lapse imaging shows that ApoE condensates exhibit liquid behavior by fusing with each other (dotted circles). (**D**) Frequency distribution of condensate volumes in RPE expressing ApoE2, ApoE3, or ApoE4. (**E**) Frequency histograms of ApoE2 condensate volumes in RPE after treatment with 1,6-hexanediol, which disrupts weak hydrophobic interactions or with vehicle (digitonin) alone. See also Supplemental Figure 4. (**B**, **D**, and **E**) Mean ± SEM, *P < 0.05, **P < 0.005, ***P < 0.0001. *n* = 15–29 cells per condition; 1-way ANOVA with Bonferroni’s posttest.

Phase-separated liquid-like condensates (a) are roughly spherical shape, (b) fuse with one another to minimize surface tension, and (c) dynamically respond to changes in the subcellular environment (46, 48–51). Live-cell imaging showed that ApoE forms spherical structures with average sphericity approximately 0.9 (Figure 4, A and B) that readily fuse and relax (Figure 4C), suggesting that ApoE2, ApoE3, and ApoE4 all segregate into liquid-like assemblies within the RPE. Although the 3 isoforms had similar disorder tendencies when analyzed by meta-predictors such as DisMeta (49) and other commonly used disorder predictors (Supplemental Figure 4, A–C), there were significantly more ApoE2 condensates with larger volumes (>0.6 μm^3^), compared with either ApoE3 or ApoE4 condensates (Figure 4D and Supplemental Figure 4, D–F). To confirm that ApoE2 forms liquid-like condensates, we treated ApoE2-expressing RPE for 2 minutes with 1,6-hexanediol, an aliphatic alcohol known to disrupt weak hydrophobic interactions that anchor biomolecular condensates (46, 48, 49). Consistent with our hypothesis of ApoE2 phase separation, we observed that 1,6-hexanediol treatment rapidly decreased both the number and the volume of ApoE2 condensates in the RPE (Figure 4E and Supplemental Figure 4G). These data show that ApoE undergoes phase separation in an isoform-specific manner in the RPE.

### Mitochondrial injury drives redox-mediated phase separation of ApoE2 to form biomolecular condensates in the RPE.

**Figure 5:**
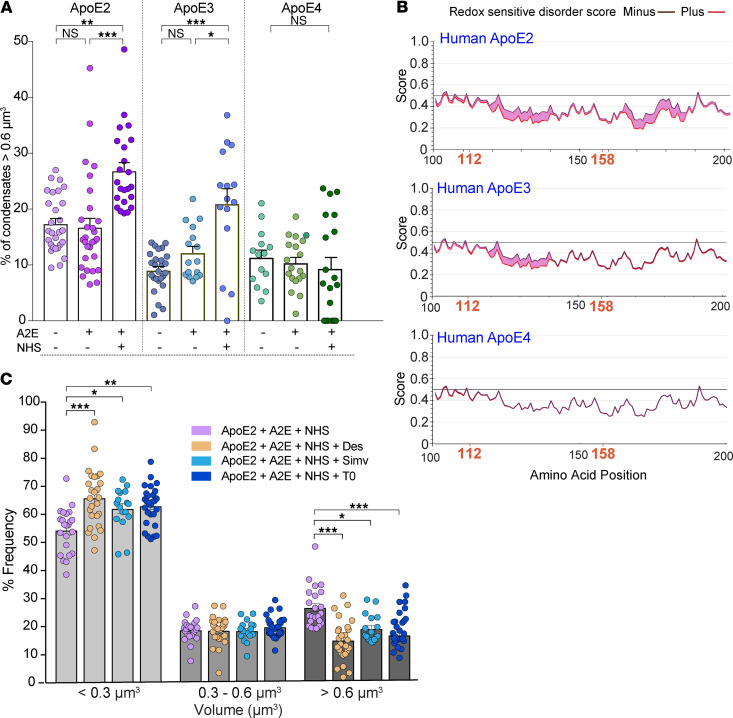
Mitochondrial injury drives redox-mediated phase separation of ApoE2 and ApoE3. (**A**) Percentage of ApoE condensates with volumes greater than 0.6 μm^3^ in RPE expressing ApoE2, ApoE3, or ApoE4 and treated with or without A2E or NHS. (**B**) IUPRED2A disorder plots for human ApoE2, ApoE3, and ApoE4. Redox state–dependent order-disorder transitions are depicted by the pink shaded area. Note increased disorder corresponding to cysteines at 112 and 158 in ApoE2 and at 112 in ApoE3. (**C**) Frequency distribution of condensate volumes in ApoE2-expressing RPE with A2E exposed to NHS and treated with simvastatin (5 μM, 16 h), T0901317 (1 μM, 16 h), or desipramine (10 μM, 3 h). Mean ± SEM, *P < 0.05, **P < 0.005, ***P < 0.0001. *n* = 15–29 cells per condition; 1-way ANOVA with Bonferroni’s posttest. See also Supplemental Figure 4.

As liquid-liquid phase separation is known to be exquisitely sensitive to intracellular environment (52), we next asked whether declining metabolic activity in ApoE2-RPE due to a loss of mitochondrial function could increase its propensity for phase separation. We analyzed condensate volumes in RPE expressing ApoE2, ApoE3, and ApoE4 under conditions known to cause cellular stress and mitochondrial damage (i.e., exposure of RPE with A2E to complement). Our data show that whereas A2E alone did not noticeably impact condensate numbers or volumes, exposure of ApoE2-RPE with A2E to NHS significantly increased the number of large (>0.6 μm^3^) condensates (Figure 5A), consistent with our mitochondrial fragmentation data (Figure 3, A–D). We also observed a small but significant increase in large condensates in ApoE3-RPE exposed to A2E and NHS, whereas ApoE4-RPE were completely resistant to this stress-induced phase separation (Figure 5A). To better understand the relationship between ApoE condensates and mitochondrial function, we used IUPRED2A (53) to predict redox state–dependent transitions in ApoE isoforms. This modeling showed that ApoE2 undergoes more order-disorder phase transitions under oxidative stress compared with ApoE3 or ApoE4 (Figure 5B and Supplemental Figure 4, H–J). ApoE2 has cysteine residues at positions 112 and 158, which can undergo reversible thiol oxidation in response to the intracellular redox environment. Pertinently, these thiol modifications enable ApoE2 to form disulfide-linked homodimers and higher order oligomers (54–56), which would explain the increase in ApoE2 condensate volumes. In agreement with this model, ApoE3, which has a cysteine at 112 and an arginine at 158, is predicted to have fewer phase transitions under oxidative stress; however, thiol modification at 112 could presumably explain the small increase in condensate formation we observed in Figure 5A. In contrast, ApoE4 has arginines at both 112 and 158 and is therefore impervious to redox state–dependent phase separation and condensate formation (Figure 5B and Supplemental Figure 4J).

Finally, to confirm the role of mitochondrial stress in driving ApoE2 phase separation, we asked if drugs that prevent mitochondrial injury after complement attack limit condensate formation. Treatment with either desipramine, simvastatin, or T0901317 significantly decreased the fraction of large ApoE2 condensates in the RPE with A2E exposed to complement (Figure 5C). Collectively, these findings identify aberrant phase separation as a powerful mechanism that could nucleate intracellular ApoE2 (and, presumably, ApoE3) condensates within the RPE, especially under conditions of declining mitochondrial health. Further, our data show that pharmacological approaches that safeguard RPE mitochondrial function limit the formation of ApoE condensates.

### Safeguarding mitochondrial health prevents pathological protein phase transitions in the RPE in vivo.

Studying the role of mitochondrial stress in ApoE phase transitions in mice is hampered by the fact that mice express a single form of ApoE. Mice with targeted replacement of human *APOE2*, *APOE3*, and *APOE4* (APOE-TR mice) have been widely used to study the role of *APOE4* in Alzheimer’s disease. However, approximately 100% of mice expressing human *APOE2* develop type III hyperlipoproteinemia, unlike humans, where only 10% of those with the *E2/E2* genotype develop the disease (57). As *APOE2* mice have increased plasma levels of chylomicrons and VLDL remnants, this will likely influence lipoprotein and cholesterol levels in the RPE and retina, which could lead to confounding results in our experiments. These issues could likely explain the differing retinal phenotypes observed in studies using the APOE-TR mice (58, 59).

**Figure 6:**
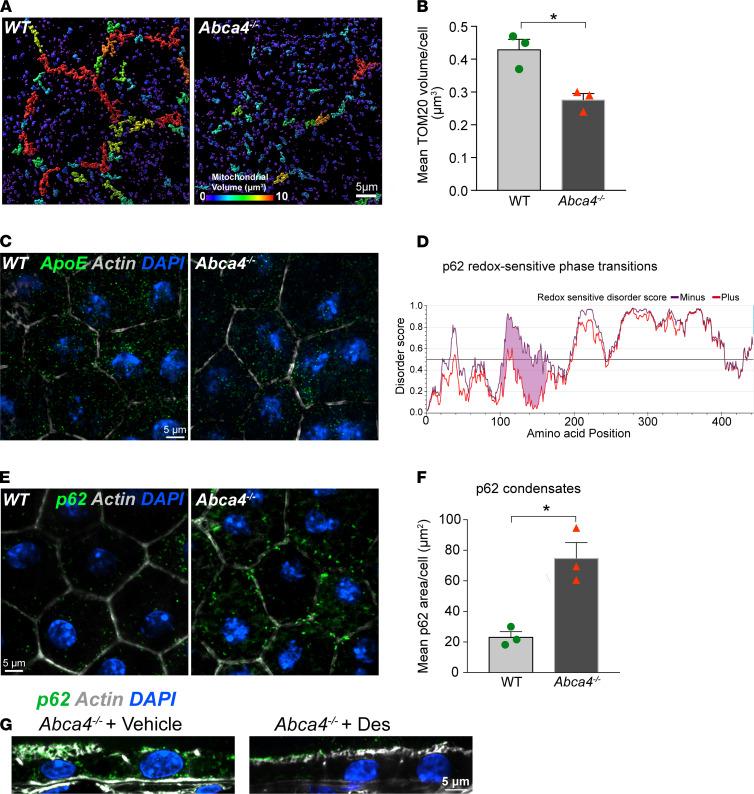
Mitochondrial injury and redox-sensitive protein phase separation in *Abca4^–/–^* mouse RPE. (**A**) Volume reconstructions of TOM20-stained mitochondria in WT and *Abca4^–/–^* mouse RPE flatmounts. Warmer colors in the color bar indicate larger volumes. (**B**) Quantification of mean TOM20 volumes from images in **A**. Mean ± SEM, *P < 0.05, *n* = 3 mice per genotype. (**C**) ApoE immunostaining (green) in WT and *Abca4^–/–^* mouse RPE flatmounts. (**D**) IUPRED2 redox-driven disorder predictions for mouse p62. (**E**) p62 condensates (green) in WT and *Abca4^–/–^* RPE flatmounts. (**F**) Areas of p62 aggregates. Mean ± SEM; *P < 0.05; *n* = 3 mice per genotype. (**G**) p62 (green) in RPE in *Abca4^–/–^* mice treated with vehicle or desipramine. (**C**, **E**, and **G**) Nuclei, DAPI (blue); actin, phalloidin (gray).

To avoid these issues, we decided to investigate redox-mediated phase transitions in the pigmented *Abca4^–/–^* mouse model of Stargardt inherited macular degeneration, which recapitulates many features of early RPE dysfunction observed in AMD, including cholesterol and ceramide accumulation, increased microtubule acetylation, and complement activation (18, 19, 32, 33). Pertinent to this study, we previously reported that lipofuscin-induced cholesterol and ceramide accumulation in *Abca4^–/–^* mice make the RPE susceptible to complement-mediated mitochondrial injury as evidenced by loss of optic atrophy 1 (OPA1), which mediates inner mitochondrial membrane fusion (19). Three-dimensional (3D) reconstructions of TOM20-stained mitochondria in mouse RPE flatmounts showed a significant decrease in mitochondrial volumes, indicative of mitochondrial fragmentation, in 6-month-old *Abca4^–/–^* mice compared with age-matched WT animals (Figure 6, A and B). Mice express a single form of ApoE with arginines at positions 112 and 158 but without the arginine at residue 61 essential for the domain interactions seen with human ApoE4 (60). Based on the lack of oxidizable cysteines at residues 112 and 158, we hypothesized that mouse ApoE would be resistant to redox state–related phase transitions seen in human ApoE2 and ApoE3. This was confirmed by IUPRED2A modeling (Supplemental Figure 4K) and immunofluorescence staining for ApoE in mouse RPE flatmounts, which showed no difference in the number or volume of ApoE puncta between WT and *Abca4^–/–^* mice (Figure 6C).

We next asked whether our model of mitochondrial redox state–mediated phase separations would be applicable to other proteins with reactive cysteines. We focused on the multifunctional protein p62/SQSTM1, which regulates autophagy, proteasomal clearance, and NRF2-mediated antioxidant response and has been implicated in AMD pathogenesis (20, 61, 62). IUPRED2A modeling predicted that p62 is highly susceptible to redox state–driven phase transitions because of cysteines at 105 and 113 (63) (Figure 6D). Immunostaining of mouse RPE flatmounts showed numerous large p62 condensates in *Abca4^–/–^* mice as a consequence of mitochondrial fragmentation compared with age-matched WT animals (Figure 6, E and F). Mean areas of p62 aggregates were 23.29 ± 4.94 μm^2^ in WT versus 74.8 ± 14.45 μm^2^ in *Abca4^–/–^* RPE. As further confirmation of the link between mitochondrial health and protein phase separation, a 1-month treatment with desipramine, which prevents complement-mediated mitochondrial injury in the RPE, decreased p62 condensates in *Abca4^–/–^* mice RPE (Figure 6G).

These data demonstrate that mitochondria regulate redox state–mediated abnormal liquid-liquid phase separation of proteins with reactive cysteines, driving the formation of intracellular biomolecular condensates in the RPE in vivo. p62 is an autophagy receptor that ferries ubiquitinated cargo to autophagosomes, and abnormal sequestration of p62 in condensates could underlie the autophagic defects reported in *Abca4^–/–^* mice RPE (33) and in AMD donor RPE (20). Our studies also demonstrate that condensate formation is reversible and identify pharmacological approaches that limit p62 condensate formation by preserving RPE mitochondrial health in the *Abca4^–/–^* Stargardt mouse model.

### Mitochondrial fragmentation correlates with ApoE and p62 aggregates in AMD donor RPE.

**Figure 7:**
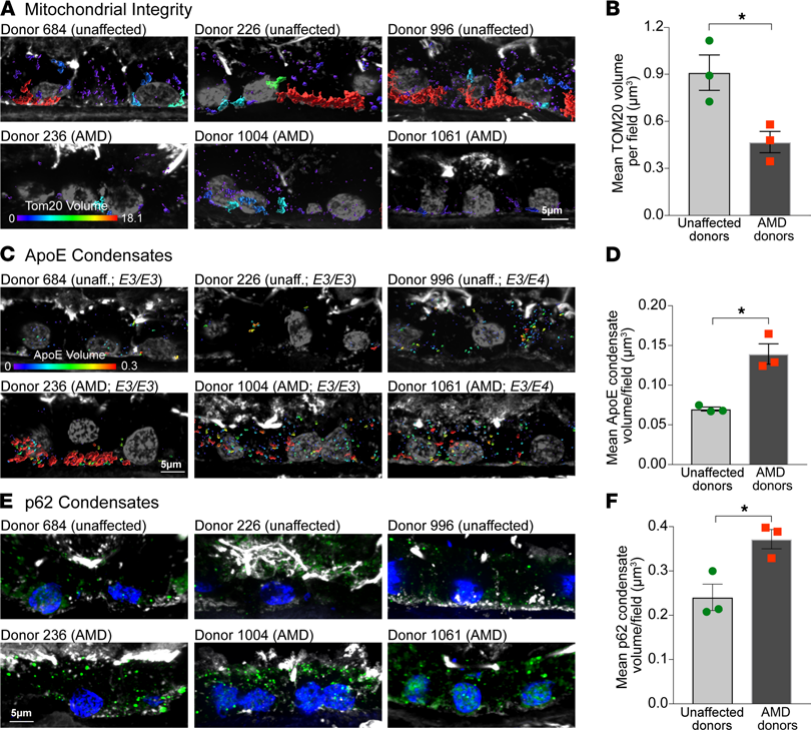
RPE mitochondrial injury correlates with ApoE and p62 condensates in AMD donors. (**A**) 3D volume reconstructions and (**B**) quantification of TOM20-labeled mitochondrial volumes in macular RPE cryosections from unaffected donors and donors with AMD. (**C**) 3D volume reconstructions and (**D**) quantification of ApoE condensates in human donor RPE. Warmer colors in the color bars in **A** and **C** indicate larger volumes. (**E**) p62 immunostaining (green) and (**F**) quantification of p62 condensates in human donors. In **A**, **C**, and **E**, phalloidin is in white and nuclei are labeled with DAPI (gray in **A** and **C** and blue in **E**). (**B**, **D**, and **F**) Data are presented as mean ± SEM, *P < 0.05, 3 donors per group. See also Supplemental Figure 5 and Supplemental Tables 2 and 3.

Building upon our in vivo data, we asked if the mechanism linking increased RPE mitochondrial dysfunction to the formation of biomolecular condensates of AMD-relevant proteins would hold in human donors with AMD. We genotyped retinal tissue from unaffected donors and donors with non-neovascular AMD for AMD-associated genes. Both control and AMD donors harbored a mixture of risk and nonrisk alleles (Supplemental Tables 2 and 3), reflecting the genetic complexity of AMD, where many individuals with risk variants do not develop the disease and vice versa (8). Volume renderings of TOM20 immunostaining in macular retinal cryosections showed significantly lower RPE mitochondrial volumes (indicative of fragmentation) in all AMD donors compared with unaffected controls (Figure 7, A and B).

Analysis of ApoE (Figure 7, C and D) and p62 (Figure 7, E and F) by immunostaining and volume reconstructions showed significantly larger ApoE and p62 condensates in AMD donor RPE compared with unaffected donors. *APOE* allele distribution in both unaffected and AMD donors was identical (2 *E3/E3* donors and 1 *E3/E4* donor each; Supplemental Table 2). The increased volume of ApoE aggregates in AMD donors with at least 1 *E3* allele correlates with the mitochondrial fragmentation in these donors and agrees with data in Figure 5, which shows that ApoE3 also undergoes redox-mediated phase separation due to the cysteine at residue 112.

These data, together with our live-cell imaging and mouse studies, provide strong support for a model in which mitochondrial injury enables phase separation of AMD-relevant proteins with reactive cysteines, leading to the formation of biomolecular condensates.

## Discussion

Deciphering the roles of AMD-associated genes in the disease process has been challenging because of limited insight into how risk variants modulate RPE homeostasis and contribute to specific features of AMD pathology (9, 64, 65). Here, using in vitro, in vivo, and ex vivo models, we show that differential cholesterol transport mediated by ApoE isoforms (ApoE2<<ApoE3=ApoE4, meaning ApoE2 is less efficient at cholesterol transport than ApoE3 or ApoE4, which are roughly equal to each other) regulates RPE susceptibility to complement attack. Further, this crosstalk between dysregulated complement and cholesterol pathways converges upon RPE mitochondria, which leads to redox-mediated phase separation of ApoE2, the AMD risk isoform. These studies help explain the risk associations of *APOE* allelic variants in AMD and demonstrate how these alleles modulate drusen biogenesis, a hallmark of AMD progression. We show that upstream therapeutic strategies that restore lipid homeostasis prevent complement activation, protect mitochondrial integrity, and limit phase separation and drusen nucleation in the RPE.

Our live-imaging data provide a basis to understand the reversed risk associations of *APOE* alleles between AMD and Alzheimer’s disease. The dynamic trafficking and efficient cholesterol transport we observed with ApoE4 agrees with studies in astrocytes (31, 66). In the brain, astrocytes deliver cholesterol to neurons in the form of ApoE lipoproteins (67), and high neuronal cholesterol is conducive for the formation of pathogenic beta-amyloid from amyloid precursor protein, suggesting that increased astrocyte-to-neuron cholesterol transport by ApoE4 is injurious to the brain (66). In contrast, in the retina, the RPE is the main biosynthetic source of ApoE, the primary hub for cholesterol trafficking into and out of the retina, and the initiator of drusen biogenesis (6, 8, 35). Cholesterol transport by ApoE occurs in association with ABCA1, and ApoE4 is shown to bind ABCA1 with greater affinity than ApoE3 in CNS astrocytes (ApoE2 was not studied) (68). Genetic variants in ABCA1 are also implicated in AMD (8), and mouse models with RPE-specific deletion of ABCA1 show RPE atrophy and retinal degeneration (17). Thus, in the context of the retina, efficient cholesterol transport mediated by ApoE4 and ABCA1 is likely to be essential for maintaining RPE cholesterol homeostasis and limiting drusen nucleation.

In the RPE, excess cholesterol and ceramide impair intracellular trafficking, leading to autophagic defects and complement-mediated mitochondrial injury (18, 19, 33). These defects were recapitulated in RPE expressing ApoE2 as a direct consequence of poor cholesterol transport and microtubule acetylation. In contrast, efficient cholesterol efflux by ApoE3 and ApoE4 preserved critical functions in RPE exposed to innate stressors (complement, lipofuscin) that are associated with AMD. Autophagic defects and mitochondrial abnormalities have been observed in AMD donor RPE (20, 22, 39, 69), and our studies now provide a molecular mechanism to explain how AMD risk variants in cholesterol genes can drive these phenotypes.

Detailed analyses of ApoE isoform dynamics led to the discovery of an exciting mechanism: ApoE liquid-liquid phase separation as an initiator of drusen biogenesis. Proteomics and cell culture studies suggest that drusen originate from the RPE (70). However, these observational studies do not address a critical question in AMD biology: how does ApoE nucleate drusen within the RPE and can we target this therapeutically? We now show that mitochondrial stress stimulates redox state–mediated phase separation of ApoE2 because of cysteines at positions 112 and 158, whereas ApoE4 (with arginines at both positions) is resistant to phase separation. ApoE3 also undergoes phase separation, albeit to a lesser extent than ApoE2, because of the single cysteine at 112. Given the low prevalence of *APOE2* and *APOE4* alleles in the general populace, these data support the multiple hit model of AMD pathogenesis in which the presence of other genetic (e.g., *CFH*, *HTRA1/ARMS2*, *C3*) or environmental (e.g., smoking, high HDL) risks (7, 8, 10, 69) could drive ApoE3 phase separation and drusen nucleation. Indeed, our data on human donors with the *E3/E3* genotype, in which the extent of RPE mitochondrial injury correlates with the number and volume of ApoE aggregates in AMD donors, lend strong support to this model.

Studies in mice confirmed the role of cysteines in mediating protein phase separation: mouse ApoE, which has arginines at 112 and 158, does not form condensates in *Abca4^–/–^* RPE, despite extensive mitochondrial fragmentation. These data also provide unexpected insight into mouse models of AMD and explain why mice do not develop bona fide drusen even in transgenic mice engineered to accumulate cholesterol in the RPE and Bruch’s membrane (71). Our mouse studies led us to another surprising discovery, that mitochondria-mediated phase separation affects not just ApoE2 but also other proteins with reactive cysteines, notably p62. Because p62 regulates multiple functions including autophagy, proteasomal clearance, and the antioxidant stress response via NRF2/KEAP1 signaling, phase separation of p62 could compromise many pathways implicated in AMD (62, 72, 73).

Liquid-liquid phase separation of proteins into biomolecular condensates has been increasingly implicated in several neurodegenerative diseases. However, modulating pathological condensate formation requires a deeper understanding of the biology that drives phase transitions (74). Our discovery of mitochondria-mediated phase separation of proteins with reactive cysteines in the RPE, coupled with the relative rarity of cysteines in the proteome (~2.3%; ref. 75), suggests that thiol-mediated selective protein phase separation could be a direct pathological consequence of mitochondrial dysfunction in the RPE. These data also identify a powerful and precise drug target for macular degenerations: rather than simply targeting drusen after they are deposited extracellularly, strategies that act upstream to preserve RPE mitochondrial health could be more beneficial in AMD by halting phase separation and consequently drusen nucleation within the RPE. In support of this, epidemiological studies show that desipramine — the ceramide-lowering drug used in this study that prevents ApoE2 and p62 phase separation — significantly decreases the incidence of early AMD (76).

The RPE is central to understanding disease pathology and developing effective therapies for AMD. These studies identify mechanisms that integrate RPE mitochondrial injury with defining but poorly understood features of AMD: complement activation, lipid dysregulation, and drusen formation. By establishing how ApoE isoforms modulate major AMD-associated biological pathways, our results provide valuable insight into the role of AMD risk variants as “tipping points” that divert the RPE from normal aging toward AMD and lay the groundwork for further research into the role of liquid-liquid phase separation and biomolecular condensates in AMD pathogenesis.

## Methods

Detailed information about reagents, sources, and experimental methods are provided in Supplemental Methods and Supplemental Table 1. Briefly, methods are given below.

### Primary porcine RPE culture.

RPE cells were isolated from freshly harvested porcine retinas using established protocols (77). For live imaging, RPE cells were plated at confluence (~300,000 cells/cm^2^) on serum-coated glass-bottom dishes (MatTek Life Sciences) as described (33, 77).

### Mice.

Both WT (129S1/SvImJ, The Jackson Laboratory) and *Abca4^−/−^* mice (Abca4tm1Ght/J, The Jackson Laboratory) on an Rpe65 Leu450 background were raised under 12-hour light/12-hour dark cycle with standard diet. Mice (~6–10 months, both sexes) were euthanized approximately 3 hours after light onset. Eyes were enucleated, and eyecups were processed for immunofluorescence staining as detailed previously (18, 19, 33). For desipramine studies, mice were i.p. injected 3 times a week for 4 weeks with either 100 μL sterile, distilled water or 10 mg/kg of desipramine hydrochloride (Enzo Life Sciences) dissolved in sterile, distilled water (18).

### Expression of EGFP or mCherry-tagged ApoE isoforms in primary RPE.

pcDNA3.1 plasmids expressing human ApoE2, ApoE3, and ApoE4 were provided by Joachim Herz (University of Texas Southwestern Medical Center, Dallas, Texas, USA) (78). EGFP- or mCherry-tagged constructs were generated by inserting the ApoE2, ApoE3, or ApoE4 cDNA into the p-EGFP-N1 or p-mCherry-N1 vector (Invitrogen, Thermo Fisher Scientific) between the XhoI and BamHI restriction sites. Sequences of the constructs were confirmed by University of Wisconsin-Madison Biotechnology Core and by Quintara. Primary porcine RPE cells were transfected with EGFP- or mCherry-tagged ApoE2, ApoE3, or ApoE4 (Amaxa Nucleofector II, Lonza). Approximately 1.5 million cells and 5 μg plasmid DNA were used for each transfection. Cells were plated at confluence (300,000 cells/sq. cm) in growth medium on serum-coated glass-bottom dishes and used for live imaging 48–72 hours later. Transfection efficiencies were approximately 30%–40% for each of the isoforms.

### Treatments and assays.

Primary RPE cultures were treated with A2E (10 μM for 6-hour, followed by 48-hour chase) to match levels found in aging human RPE (33, 79). Other reagents used were the ASMase inhibitor desipramine (10 μM, 3 hours; MilliporeSigma), the LXRα agonist T0901317 (1 μM for 16 hours; Cayman Chemicals), and the lipophilic statin simvastatin (5 μM for 16 hours; Cayman Chemicals) (33, 80). Cholesterol levels were measured from RPE lysates using the Amplex Red Cholesterol Assay Kit (Thermo Fisher Scientific) (33).

### Live imaging and analysis of ApoE trafficking.

Primary porcine RPE cultures expressing mCherry-tagged ApoE2, ApoE3, or ApoE4 were imaged using the Andor Revolution XD with 100×/1.49 NA Apo TIRF objective (Nikon) for approximately 50 frames at 37°C. Trafficking data were collected from 3 separate transfections for a total of at least 17–23 movies captured per condition with the same laser power, exposure, and electron-multiplying gain settings for all conditions. For analysis of trafficking parameters, ApoE-labeled vesicles were subjected to surface reconstruction using the Surfaces and Tracks modules, and track length, track displacement, and track lifetimes were calculated using Imaris v 8.7.4 (Bitplane).

### Live imaging and analysis of autophagosome biogenesis and trafficking.

Primary porcine RPE cultures expressing mCherry-tagged ApoE2, ApoE3, or ApoE4 were transduced with LC3B-GFP (Thermo Fisher Scientific) for 16–24 hours. Autophagosome trafficking was monitored by live imaging and analysis of autophagosome numbers and trafficking parameters was performed using Imaris as previously described (33).

### Live imaging of mitochondrial dynamics.

Primary porcine RPE cultures expressing mCherry-tagged ApoE2, ApoE3, or ApoE4 with or without A2E (10 μM for 6 hours followed by a 48-hour chase in fresh culture medium) were exposed to 10% NHS for 10 minutes (19). Cells were incubated with 0.2 μM MitoTracker Deep Red (Thermo Fisher Scientific) for 15 minutes at 37°C and imaged immediately. For analysis of mitochondrial volume, MitoTracker-labeled mitochondria were subjected to surface reconstruction in Imaris, and automated segmentation by color-coding based on the volume of the connected components was used for 3D surface rendering of mitochondria.

### Analysis of ApoE condensate dynamics.

Primary porcine RPE cultures expressing mCherry-tagged ApoE2, ApoE3, or ApoE4 were imaged live using the same imaging conditions and parameters established for imaging ApoE trafficking to capture volume, fusion, and fission events. In some experiments, RPE cultures expressing mCherry-tagged ApoE2 were treated with 0.5% 1,6 hexanediol (MilliporeSigma) in digitonin or digitonin alone (vehicle) to disrupt weak hydrophobic interactions or with T0901317, simvastatin, or desipramine as above. Condensate volumes were quantified by 3D surface reconstruction and measuring mCherry fluorescence intensity per pixel of each condensate per *z*-plane of the confocal image. Histograms of number and volumes of ApoE condensates were constructed using Matlab (MathWorks).

### Genotyping and immunostaining of human donor globes.

Globes from unaffected human donors and donors diagnosed with AMD were obtained from Lions Gift of Sight (Saint Paul, Minnesota, USA) with deidentified demographics (Supplemental Table 2) and fundus photographs. DNA was extracted from frozen donor retinal tissue (PureLink Genomic DNA Kit, Thermo Fisher Scientific) and amplified by PCR using specific primers before genotyping (Quintara; refs. 8, 22, 81; Supplemental Table 3). Cryosections of donor retinas were stained with antibodies against TOM20 (1:200, sc-11415, Santa Cruz Biotechnology), ApoE (1:200, GTX100053, Genetex), or p62/SQSTM1 (1:200, NBP148320, Novus Biologicals). Sections were labeled with Alexa Fluor–conjugated secondary antibodies (A-31571, A-31573, A-21470, Thermo Fisher Scientific), rhodamine-phalloidin, and DAPI to label the actin cytoskeleton and nuclei, respectively. Sections were imaged by spinning disc confocal microscopy (Nikon CSU-X1 dual camera platform spinning disc confocal microscope) and analyzed as described below.

### Quantification of mitochondrial volumes and protein condensates.

Images of immunostaining were analyzed using Imaris 9.6 (Bitplane). Images were subjected to Gaussian filtering and background subtraction. Surface reconstructions were made with the “Surfaces” module. Mean volumes and areas were exported from the statistics tab and analyzed in Microsoft Excel and GraphPad Prism.

### Statistics.

Data were analyzed using either 1-way ANOVA with Bonferroni’s posttest or multiple 2-tailed *t* test with Welch’s correction for unequal variances (GraphPad Prism). Unless otherwise stated, data are presented as mean ± SEM of 3 or more independent experiments, with 3 or more replicates per condition per experiment. *P* < 0.05 was considered significant.

### Study approval.

All animal procedures were reviewed and approved by the IACUC at the University of California, San Francisco.

## Author contributions

NLC, LXT, KAT, and AL designed the study. NLC, LXT, and KAT performed the experiments. KAT, LXT, GR, and CJG generated primary RPE cultures. NLC, GR, and CJG processed human donor tissues. TT and NLC performed the genotyping. NLC, LXT, KAT, TT, and AL analyzed the data. GR and TT critically reviewed the manuscript. NLC, LXT, CJG, KAT, and AL wrote the manuscript. The order of the co–first authors in the author list was decided as follows: NLC was involved in the study from the initial stages and LXT helped complete it.

## Supplementary Material

Supplemental data

Supplemental Video 1

Supplemental Video 2

Supplemental Video 3

Supplemental Video 4

Supplemental Video 5

Supplemental Video 6

Supplemental Video 7

Supplemental Video 8

## Version 1 04/06/2021

In-Press Preview

## Version 2 05/10/2021

Electronic publication
